# Clinical Features and Sleep Analysis of Chinese Patients with Fatal Familial Insomnia

**DOI:** 10.1038/s41598-017-03817-3

**Published:** 2017-06-15

**Authors:** Liyong Wu, Hui Lu, Xianling Wang, Jia Liu, Chaoyang Huang, Jing Ye, Cuijiang Li, Jun Lu, Yuping Wang, Jianping Jia, Shuqin Zhan

**Affiliations:** 1 Department of Neurology, Xuan Wu Hospital, Capital Medical University, Beijing, China; 2 Department of Neurology and Division of Sleep medicine, Harvard Medical School and Beth Israel Deaconess Medical Center, Boston, MA 02115 USA

## Abstract

This study aimed to examine clinical features, sleep, abnormal sleep-wake transition and non-sleep disturbances as well as lab tests in Chinese fatal familial insomnia (FFI) subjects. Patients with confirmed clinical and laboratory diagnosis of FFI have been retrospectively reviewed. The clinical features and the results of the complementary tests, including polysomnography (PSG), brain imaging and genetic analysis, were used. Two male and three female patients were recruited in this study. Three of the five patients had more comprehensive family medical records. The most typical clinical manifestations in all 5 patients were sleep disturbances, including insomnia, laryngeal stridor, sleep breath disturbance, and sleep-related involuntary movements. PSG of all these five cases showed reduction in total sleep time, sleep fragmentation, abnormal short non-rapid eye movement - rapid eye movement (REM) cycling, REM sleep reduction or loss, and REM sleep instruction in wakefulness. Patient 2's emission tomography scan demonstrated a reduction in glucose uptake in the left thalamus and bilateral inferior parietal lobe. In summary, Chinese FFI patients are typically characterized by organic sleep related symptoms, rapidly progressive dementia and sympathetic symptoms. We propose that structural damages in the thalamus and cortex are mostly responsible for clinical manifestations of FFI.

## Introduction

Fatal familial insomnia (FFI) is a rare prion disease first described by Lugaresi *et al*., in 1986^1^. The prevalence of FFI is one case per a million population per year, with only about 57 cases in 27 kindreds have been reported worldwide^2^.

FFI is an autosomal dominant disease that harbors a missense GAC to AAC mutation at codon 178 of the *PRNP* prion protein gene located on chromosome 20, along with the presence of the methionine polymorphism at position 129 of the mutant allele^3, 4^. Pathologically, FFI is characterized by predominant thalamic degeneration especially in the medio-dorsal and anterio-ventral nuclei^5, 6^. Phenotypic variability is a perplexing feature of FFI. FFI is mainly characterized by prominent sleep impairment combined with neuropsychiatric disorders, dysautonomia and motor dysfunction^7^. Polysomnography (PSG) recordings document the biophysiological changes that occur during sleep. In FFI, PSG has disclosed a severely reduced total sleep time, reduced rapid eye movement (REM) sleep and abnormal stage shifts. In some cases, FFI was characterized by the lack of REM-associated muscle atonia and the presence of jerky activity of limb muscles and irregular breathing^8^.

Irregular breathing, hypnic jerks, propriospinal myoclonus at the wake-sleep transition, and quasi-purposeful limb gestures are considered to be characteristic features of FFI^6, 7^. Further, husky voice was reported in 22% of FFI patients in Germany^9^. Nevertheless, the mechanism and diagnostic value of sleep related respiratory disturbance and movement disorder in FFI have not been studied thoroughly yet. Here, we have analyzed the clinical manifestations and biological changes, especially the sleep related symptoms, PSG and brain imaging results of five Chinese patients diagnosed with FFI. This study aims to understand the pathogenesis of sleep related disorders in FFI.

## Results

### Clinical characteristics and neurological features

A total of five FFI patients of Chinese Han decent (2 males and 3 females) were enrolled in the current study. The median age at FFI onset was 46.4 years (from 19 to 62 years) and the duration of the course of FFI ranged from 8 to 18 months. The most prominent clinical manifestations in all five patients was sleep disturbances that included insomnia, laryngeal stridor, sleep breath disturbance, oneiric or stuporous episodes with hallucinations and confusion, and sleep-related involuntary movements (such as hypnic jerks, restless sleep with frequent changes in body position and twitchy non-purposeful movement of limbs). Furthermore, we have observed the development of a rapidly progressive dementia (RPD) along with psychiatric symptoms in all patients. MMSE (mini-mental state examination) scores of case 1 and case 2 were only 4 (Normal reference ≥ 20), CDR (clinical dementia rating) of case 5 was 2. Other patients had severe dementia and they could not complete cognitive scales. Progressive sympathetic symptoms i.e. hypertension, sweating, tachycardia, and irregular breathing were also presented by the patients enrolled in this study. Ataxia, pyramidal sign, extrapyramidal sign and dysarthria were also observed. The main clinical features and neurological features of these five cases are summarized in Table 1.

**Table 1:** Clinical characteristics of the five FFI patients.

	Case 1	Case 2	Case 3	Case 4	Case 5
Gender	M	F	F	F	M
Age at onset (years)	62	60	19	36	55
Onset to diagnosis duration (months)	3	5	3	8	11
Onset to death duration (months)	12	11	8	10	18
**Cluster A—sleep-related symptoms**					
Insomnia	+	+	+	+	+
Sleep-related involuntary movements	+	+	+	+	+
Sleep-related dyspnea	+	+	+	+	+
Laryngeal stridor	+	+	+	+	+
**Cluster B—neuropsychiatric symptoms**					
RPD	+	+	+	+	+
Psychiatric symptoms	+	+	−	+	+
Ataxia	−	+	+	+	+
Pyramidal sign	−	−	+	+	−
Parkinsonism	−	−	−	−	+
**Cluster C–progressive sympathetic symptoms**					
Autonomic symptoms					
Hypertension	+	+	−	+	−
Sweating	+	+	−	+	+
Tachycardia	+	−	+	+	−
Irregular breathing	−	−	+	−	−

RPD: rapidly progressive dementia. +: symptom/sign observed, −: symptom/sign not observed. Psychiatric syndromes included hallucination, personality change, depression, anxiety, aggressiveness, disinhibition, listlessness etc. Autonomic signs include hypertension, sweating, tachycardia, irregular breathing etc. Sleep-related involuntary movements included frequent changes in the body position and twitchy non-purposeful movements of limbs during sleep.

### Familial medical history and genetic analysis

Patients 1 and 2 were siblings, and interviews with family members have noted that their mother and an elder brother had similar FFI symptoms. Furthermore, the mother of Patient 5 died at the age of 70 years old due to rapid progressive dementia (RPD). The other two patients (Patients 3 and 4) had no known family history.

**Figure 1:**
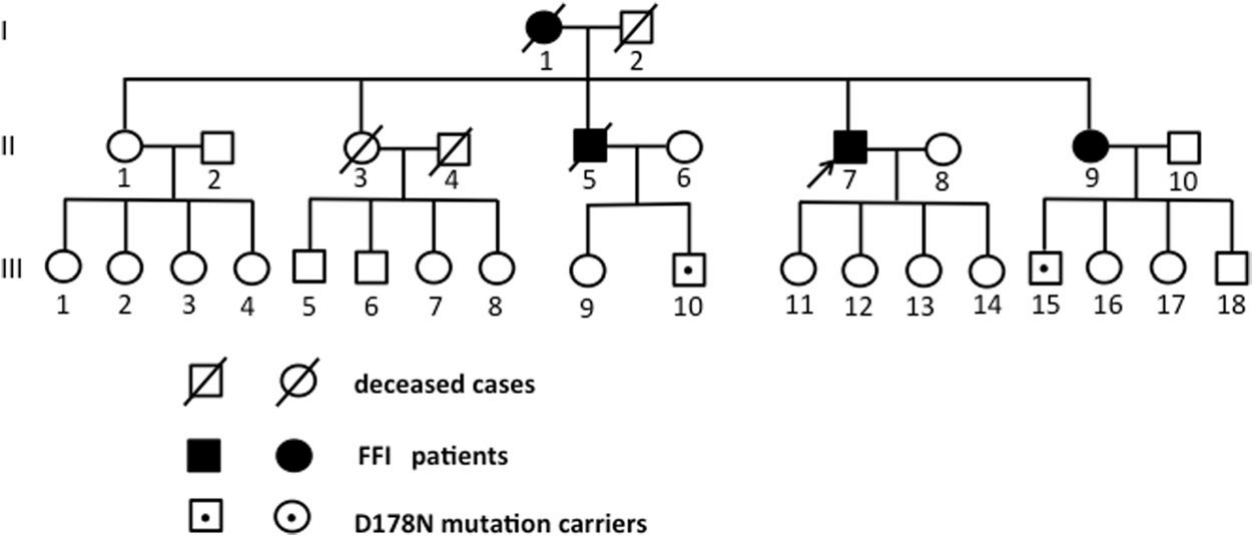
The pedigree of case 1(II-7) and case 2(II-9). II-7 is the proband of this pedigree (arrow). II-7 and II-9 are respectively Patient 1 and Patient 2 who carried *PRNP* D178N mutation. Additionally, seven family members were screened for *PRNP* gene mutation: II-6, III-6, III-11, III-13, and III-14 were healthy participant free of *PRNP* D178N mutation; III-10 and III-15 were clinically healthy carriers of the *PRNP* D178N mutation. Other family members were not screened due to lack of consent or prior death.

Gene sequencing of DNA extracted from the peripheral blood leukocytes revealed that all five patients had a D178N mutation with methionine/methionine homozygosity at the polymorphic 129 codon of the *PRNP* gene. Seven family members of Patients 1 and 2 were screened for *PRNP* gene mutation. Other family members were not screened due to lack of consent or prior death. Results of the screened family members demonstrated that the healthy control subjects number II-6, III-6, III-11, III-13, and III-14 were free of the *PRNP* D178N mutation and subjects number III-10 and III-15 were clinically healthy carriers of the *PRNP* D178N mutation (Fig. 1). Patient 3’s mother was also screened for *PRNP* gene mutation, and she was also a carrier of the D178N mutation without displaying the clinical FFI symptoms (data not shown).

### The expression of 14-3-3 protein, EEG analysis, and brain imaging

**Figure 2:**
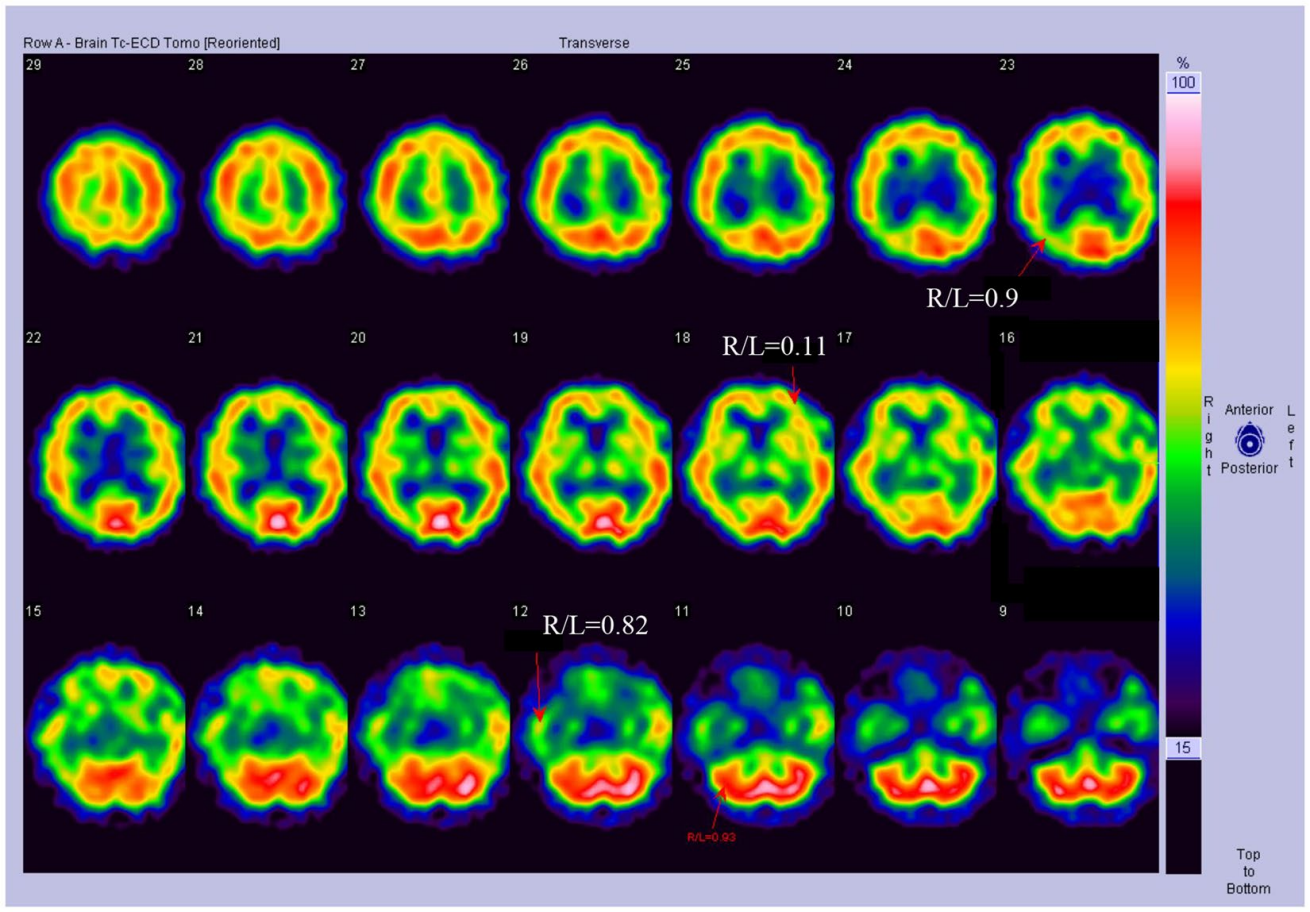
SPECT analysis of FFI patients. Patient 1 **S**PECT image showing reduced blood flow perfusion in bilateral temporal lobes, bilateral basal ganglia, and bilateral thalamus.

**Figure 3:**
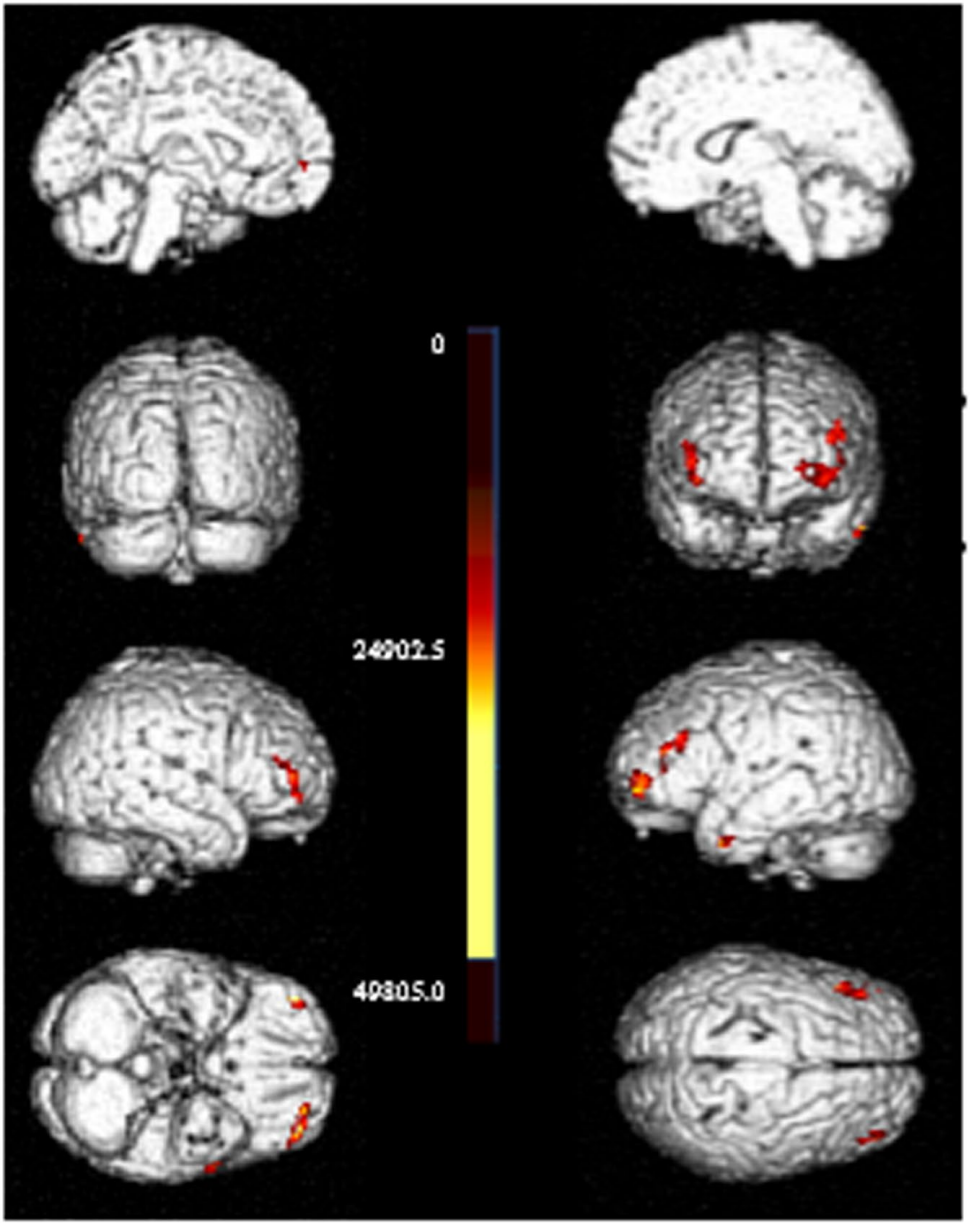
PET analysis of FFI patients. PET image for Patient 2 indicating reduced glucose metabolism in the bilateral frontal and parietal lobes and left thalamus.

ELISA results confirmed that all patients were negative for the CSF 14-3-3 protein, consistent with previous reports indicating that 14-3-3 protein is usually normal or nonspecifically changed in FFI^10^. EEG showed diffuse slow waves without periodic spike discharges in the five patients, and the brain MRI did not reveal any abnormalities (data not shown). SPECT performed on Patient 1 indicated a decline in blood perfusion of the bilateral thalamus, basal ganglia, and the medial temporal lobe (Fig. 2). Furthermore, MRS analysis for Patient 1 showed a declined level of N-aceytl aspartate (NAA) in the left thalamus (data not shown). Patient 2 underwent a PET scan, which demonstrated decreased glucose uptake in the left thalamus and bilateral inferior parietal lobe (Fig. 3).

### PSG analysis shows signs of sleep-related respiratory disturbance and leg-movement

All five patients enrolled in this study underwent PSG. The results demonstrated an early reduction in the sleep spindles and K complexes, a drastic reduction in the total sleep time by about 50% (average total sleep amounts = 212.3 ± 26.6 minutes), and decreased sleep efficiency. In addition, disruptions of the normal cyclic sleep organization, especially the loss or decreased ratio of REM phase and prolonged sleep latency of REM were also detected. In Patient 3, REM sleep occurred directly from wakefulness. The PSG indices for sleep efficiency and sleep organization are listed in Table 2. Moreover, PSG identified sleep apnea syndrome in all FFI patients, characterized by obstructive apnea/hypopnea and laryngeal stridor in the NREM stage. We observed involuntary movements in these five patients during sleep, mostly during the NREM stage 2. We did not detect PLMS. The PSG indices for sleep-related respiratory disturbance and leg-movement are listed in Table 3.

**Table 2:** Sleep efficiency and sleep organization indices for the five FFI patients.

	Case 1	Case 2	Case 3	Case 4	Case 5	Ref.
Time in bed (min)	509.0	513.0	593.5	549.5	614.0	419–464
Total sleep time (min)	167.0	269.5	145.5	277.5	202.0	402–449
Sleep efficiency (%)	32.8	52.5	24.5	50.5	32.9	85–98
NREM Stage 1 (%)	0.6	5.2	3.8	4.1	5.0	2–5
NREM Stage 2 (%)	32.9	24.1	68.0	31.4	59.2	45–55
NREM Stage 3 (%)	66.5	70.7	16.2	61.3	25.5	20–25
REM (%)	0	0	12.0	3.2	10.4	20–25
Latency to REM stage (min)	—	—	339.0	208.5	117.5	90–110

Ref. stands for reference value range; REM: rapid eye movement; NREM: Non- rapid eye movement.

**Table 3:** Sleep-related respiratory disturbance and leg-movement indices of the five FFI patients.

	Case 1	Case 2	Case 3	Case 4	Case 5	Ref.
BMI	23	20.8	19.2	19.5	20.5	18–24
Baseline oxygen saturation	95	97	97	97	94	
Minimum oxygen saturation	83	89	86	91	76	
AHI	10.8	7.8	41.6	4.3	18.7	<5
Obstructive apnea	+	+	+	+	+	−
Laryngeal stridor	+	+	+	+	+	−
NREM limb movements	425	951	225	557	229	−
REM limb movements	0	0	0	2	4	−
PLMS index	0	0	0	0	0	<5

AHI: apnea/hypopnea index; PLMS: periodic leg movement in sleep; +: symptom/sign observed; −: symptom/sign not observed.

### Hypnogram analysis

**Figure 4:**
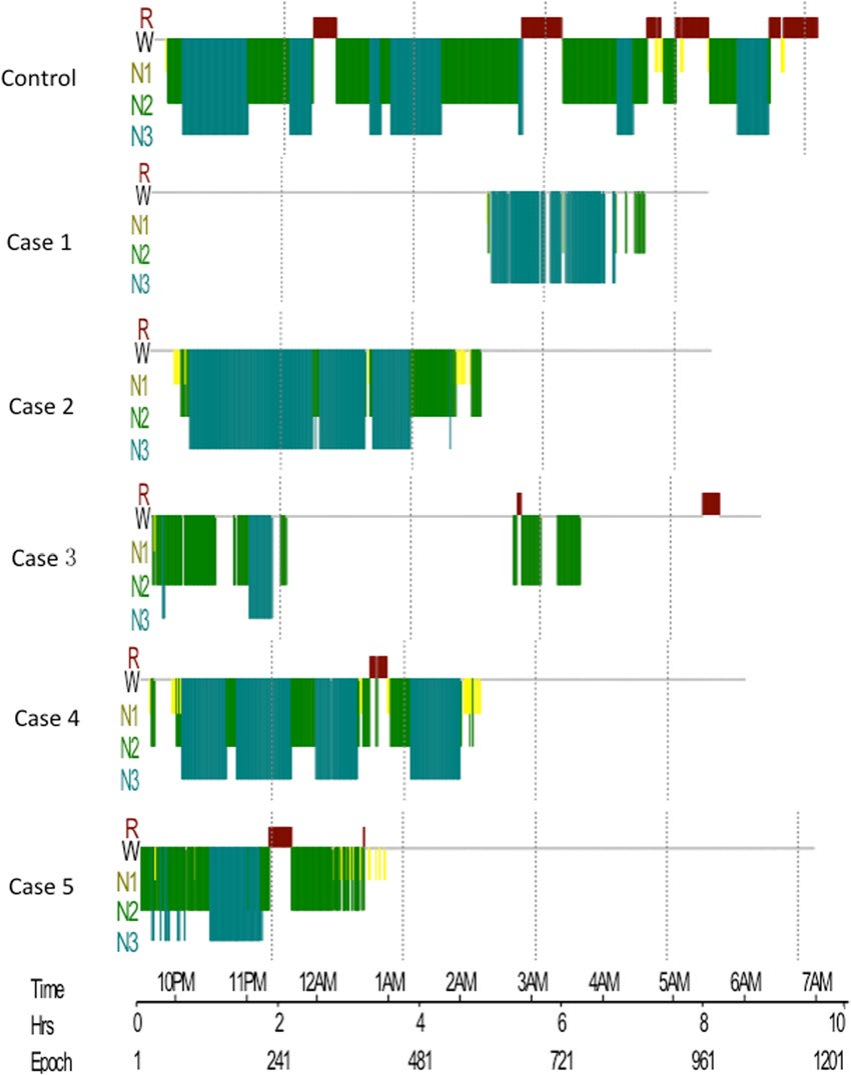
Hypnogram of a healthy control and the five FFI patients. The 5 FFI patients presented with decreased sleep efficiency and disruption of the normal cyclic sleep organization (horizontal axis in sleep hours). W: wake, R: REM, N1-3: NREM sleep stages.

Finally, the hypnogram analysis for the five FFI patients has revealed a decreased sleep efficiency and disruption of the normal cyclic sleep organization. In contrast, the healthy control participants successfully completed several sleep cycles (Fig. 4).

## Discussion

FFI is a rare fatal genetic disease that affects both genders equally. The mean age at disease onset is approximately 50 years (range, 21–62 years), and the FFI duration varied from 7 to 72 months^7, 11^. Increased insomnia and sleep-wake cycle disruption are the clinical hallmarks of FFI. In this study, we reported the clinical aspects as well as the analysis of sleep cycle and sleep-related disturbances for five Chinese Han patients. We have compared symptoms of Chinese FFI patients with FFI patients reported from other countries, especially those from Italy and Germany, and no major differences were found. Patient 3 had an early onset of FFI at the age of 19 years. To the best of our knowledge, this is the youngest FFI patient ever reported. Genetic analysis of the *PRNP* gene confirmed that Patient 3’s mother was a healthy carrier of the D178N mutation, suggesting that *PRNP* gene mutation may have an autosomal dominant trait with incomplete penetrance.

The alterations in the sleep cycle in the five FFI cases were investigated by PSG recording. All patients showed a declined sleep efficiency and disruption of the cyclic sleep organization. The recording revealed abortion of the REM sleep or reduction of REM and increased latency to reach the REM stage. Furthermore, PSG results revealed an early reduction in the sleep spindles and K complexes in our patients, and these results came in accordance with previous studies^1–3^. Sleep spindles and K-complexes indicate the transition from light to deep NREM sleep. It has been confirmed that sleep spindles are produced by the thalamus^12^. Therefore, the loss of the sleep spindles and K-complexes suggests a decline in thalamic function^8^.

In this study, we discovered characteristic sleep features that were rarely reported in previous FFI cases, i.e., sleep apnea syndrome, laryngeal stridor, and sleep-related involuntary movements. In good agreement with these clinical symptoms, PSG recorded obstructive apnea/hypopnea, laryngeal stridor, and NREM stage 2 involuntary movement in the five patients enrolled in this study. Therefore, it is plausible to speculate that active laryngeal narrowing during inspiration led to the development of obstructive apnea/hypopnea and laryngeal stridor in patients with FFI. The paralysis of the posterior cricoarytenoid muscle was reported to cause passive laryngeal narrowing producing the inspiratory stridor^13^. In the five cases enrolled in this study, we did not observe PLMS events; however, we noticed involuntary limb movements, especially in NREM stage 2. This result came in accordance with previous studies that reported hypnic jerks, quasi-purposeful limb gestures, and propriospinal myoclonus atypical to the NREM stage^2, 6, 7^. It was proposed that the abnormal involuntary movements during sleep were attributed to the thalamus^14, 15^.

The histopathological hallmark of FFI is severe neuronal loss and deposition of the pathogenic prion protein (PrP^Sc^)^16^. Previous postmortem neuropathological studies revealed severe atrophy of the thalamus, particularly in the anteroventral and dorsomedial thalamic nuclei, and the inferior olives^5, 6^. Additionally, moderate atrohpy of the cortex and basal ganglia were previously observed in FFI cases^17^. In this study, routine imaging of the brain did not reveal characteristic FFI features except for variable degrees of cerebral and cerebellar atrophy. Flourodeoxyglucose (^18^F) PET scan uncovered a profound bilateral thalamus with less pronounced cingular hypometabolism compared with the FFI signature image^18, 19^. In accordance with previously published data, the SPECT scan of Patient 1 and PET scan of Patient 2 in our study confirmed hypometabolism in the thalamus. Bar *et al*. previously reported that hypometabolism was confined to the thalamus in the earlier stages of the disease^18^.

Previous histopathological studies indicated severe neuronal loss in the thalamus^17^. Therefore, it was hypothesized that the dysregulation of the sleep-wake cycle is attributed to thalamic lesions^20^. However, animal studies showed that the thalamus is not critical or necessary for sleep-wake regulation^21^. Although thalamic strokes may cause hypersomnance, further analysis suggested that those strokes often extend to the hypothalamus and midbrain, which interferes with the ascending arousal projections. We speculate that basal ganglia-cortex circuits may also be involved in the regulation of the sleep-wake cycle. In this study, SPECT and PET scans revealed that basal ganglia and the cerebral cortex were affected in FFI. Furthermore, results obtained from rat models with induced lesions revealed that the basal ganglia are necessary for sleep architecture^22, 23^.

In conclusion, in this study, we analyzed the clinical features and sleep patterns of five FFI cases. We observed decreased sleep efficiency and disruption of the normal cyclic sleep organization. Nevertheless, this study had a few limitations like the small number of examined cases and the absence of histopathological data. In future studies, we will include a larger number of FFI cases, perform a detailed histopathological study, and use MRS, SPECT, and PET scans to elucidate the mechanism of FFI.

## Materials and Methods

### Ethics Statement

This study protocols outlined in this manuscript were approved by the Ethics Committee and local Institutional Review Board (IRB) of Xuan Wu Hospital, Capital Medical University, Beijing. All methods and experiments were performed in accordance with the relevant guidelines and regulations. All patients and healthy participants enrolled in the study signed an informed written consent specifically approved for this study prior to the study commence.

### Subjects

This was a retrospective study that included five Chinese patients of Han descent who had been diagnosed with FFI and admitted to Xuan Wu Hospital (Beijing) from 2009 to 2014. The patients were diagnosed according to the FFI diagnosis criteria mentioned previously^9^. The clinical and phenotypic features of FFI were recorded for each patient at the time of admission.

### Genetic and biochemical analysis for 14-3-3 protein epxression and PRNP gene mutation

Blood samples were obtained from all patients as well as several healthy control family members of the sibling patients #1 and #2 and patient’s #3 mother. *PRNP* gene mutation was detected in DNA isolated from the peripheral blood leukocytes as previously detailed^16, 24^. The 14-3-3 protein analysis was performed on cerebrospinal fluid samples by enzyme-linked immunosorbent assay (ELISA; Beijing TIANGEN) following the manufacturer’s protocol as described previously^25^.

### EEG and brain imaging

EEG and brain magnetic resonance imaging (MRI) were performed for all five patients. In addition, Patient 1 underwent brain single photon emission computed tomography (SPECT) to detect blood flow perfusion and magnetic resonance spectrum (MRS). Patient 2 underwent a FDG positron emission tomography (PET) scan to illustrate the glucose metabolism of brain.

### PSG analysis

Each patient underwent standard PSG (E-Series, Compumedics Limited, Abbotsford, Australia) study. The sleep-related laryngeal stridor, sleep breath disturbance, and involuntary movements were recorded. Sleep scoring was carried out for 30-second epochs according to AASM scoring criteria 2012. Next, sleep structure, sleep efficiency (total sleep time/total time in bed ×100%), arousal, periodic limb movement in sleep (PLMS) indices (AI and PLMS-I) (number of arousals and PLMS per hour of sleep), and the calculated AHI (apnea + hypopnea index) were evaluated.

### Hypnogram analysis

Sleep stages (wake, N1, N2, N3, and REM sleep) were scored offline according to the American Academy of Sleep Medicine (AASM) scoring criteria by visual and spectral inspection of 30-second EEG epochs^26^.

### Statistical analysis

In this study, the SPSS software was used to evaluate statistical significance. Differences in measured variables were assessed by using Student’s *t* test or Wilcoxon rank-sum test with nonparametric data. Results were considered statistically significant at *P* < 0.05.
